# Syndrome de Costello: à propos d'une observation

**Published:** 2012-07-04

**Authors:** Mariam Tajir, Patricia Fergelot, Gwenaelle Lancelot, Benoit Arveiler, Siham Chafai Elalaoui, Didier Lacombe, Abdelaziz Sefiani

**Affiliations:** 1 Centre de Génomique Humaine, Faculté de Médecine et de Pharmacie, Université Mohamed V Souissi, Rabat, Maroc; 2 Département de génétique médicale – Institut National d'Hygiène, Rabat, Maroc; 3 Service de génétique médicale, CHU Hôpital Pellegrin, Bordeaux, France

**Keywords:** Syndrome de Costello, gène HRAS, Costello syndrome, HRAS gene

## Abstract

Le syndrome de Costello appelé également syndrome Facio-cutanéo-squelettique est une anomalie rare du développement d'origine génétique de transmission autosomique dominante. Sa prévalence est inconnue mais environ 250 cas ont été rapportés dans la littérature. La majorité des cas sont sporadiques. Les principaux signes sont une dysmorphie faciale caractéristique, un retard mental, un retard de croissance, un cutis laxa, une malformation cardiaque et des papillomes péri-orificiels. Sur le plan génétique, ce syndrome est dû à des mutations au niveau du gène HRAS qui est un oncogène de la famille Ras. Ceci est à l'origine de la prédisposition de ces patients au développement de tumeurs malignes. Le pronostic dépend de la sévérité de la cardiopathie et de la survenue de tumeurs malignes. Les principaux diagnostics différentiels incluent le syndrome de Noonan et le syndrome cardio-facio-cutané. Nous rapportons dans ce travail, une patiente âgée de 13 ans qui présente des signes cliniques du syndrome de Costello. L'analyse moléculaire du gène HRAS a mis en évidence la mutation c.34G>A; p.Gly12Ser de novo au niveau de l'exon 1 de ce gène. Nous montrons à travers cette observation l'intérêt de l’étude moléculaire pour confirmer le diagnostic du syndrome de Costello et pour le conseil génétique de la famille.

## Introduction

Le syndrome de Costello est un syndrome polymalformatif complexe d'origine génétique. Il se manifeste dans les premiers mois de la vie par un retard de croissance, une dysmorphie faciale caractéristique, des anomalies cutanées et cardiaques et par la suite un déficit intellectuel de sévérité variable. Le syndrome de Costello se transmet selon un mode autosomique dominant. Son diagnostic est essentiellement clinique. La prévalence de ce syndrome est inconnue, mais environ 250 cas ont été rapportés dans la littérature [1]. Des mutations de novo du gène HRAS ont été incriminées dans la majorité des cas du syndrome de Costello. Ce gène est un oncogène de la famille Ras, à l'origine de la prédisposition de ces patients au développement de tumeurs malignes [2].

Nous présentons dans ce travail, l'observation d'une patiente marocaine qui présente un phénotype clinique du syndrome de Costello et chez qui l'analyse moléculaire du gène HRAS a mis en évidence la mutation responsable. A travers cette observation, nous présentons l'intérêt de l'expertise clinique et de l’étude moléculaire dans le diagnostic des syndromes génétiques rares et pour le conseil génétique des patients et leurs familles.

## Patient et observation

Nous rapportons le cas d'une patiente âgée de 13 ans, non consanguine, deuxième d'une fratrie de deux, adressée à la consultation de génétique médicale pour un retard de croissance. Il n'y a pas de cas similaires dans la famille. L'histoire anté-néonatale a été marquée par la présence d'un hydramnios pendant le troisième trimestre de la grossesse, un poids de naissance de 4600 g, la notion d'hypotonie néonatale ainsi que des épisodes d'hypoglycémie au cours des premières semaines de la vie. Le développement psychomoteur et la croissance de notre patiente ont été marqués par une mauvaise prise pondérale, un retard statural, un retard des acquisitions psychomotrices, des troubles alimentaires à type de troubles de la déglutition (alimentation par sonde nasale jusqu’à l’âge de 4 ans), avec un retard mental modéré et un retard de l’âge osseux. Il s'agit d'un enfant calme et très sociable.

**Figure 1:**
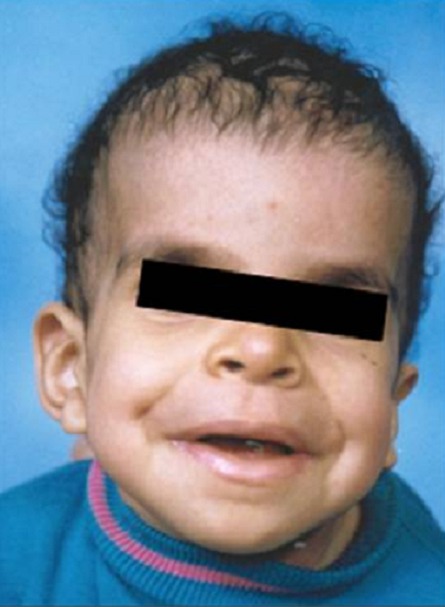
Photographie de la patiente à l’âge de 2 ans

**Figure 2:**
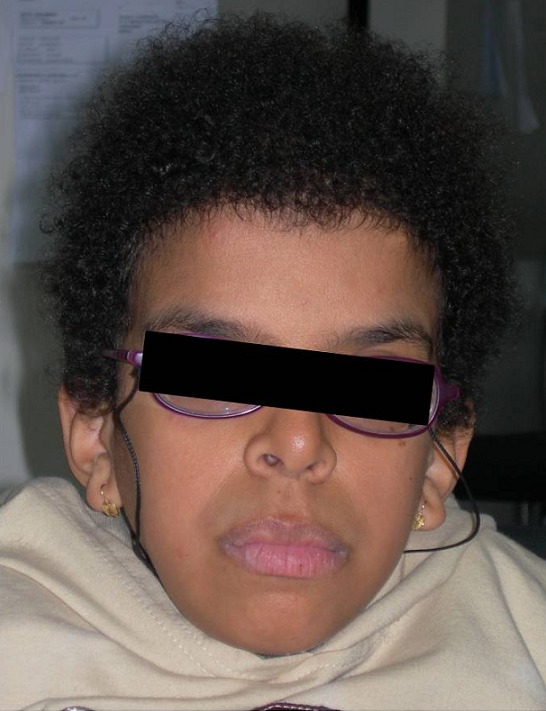
Photographie de la patiente à l’âge de 13 ans

**Figure 3:**
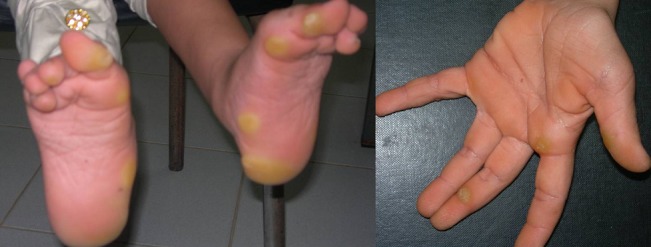
Photographies montrant l'hyperkératose palmo-plantaire

L'examen clinique actuel trouve une taille de 130 cm (-4DS), un poids de 27 kg (-3DS) et un périmètre crânien à 55 cm. Notre patiente présente une dysmorphie faciale faite d'un strabisme convergent, une myopie, des replis épicanthiques, des narines antéversées, des oreilles bas implantées en rotation postérieures, des lobes d'oreilles larges, une macrostomie, des lèvres épaisses, une malimplantation dentaire et des cheveux éparses et frisés (Figure 1, Figure 2). Elle présente également des anomalies cutanées, notamment une hyperkératose palmo-plantaire, des plages d'hyperpigmentation, une peau lâche et des papillomes périnasaux (Figure 3). L'examen clinique trouve également un cou qui est court, un pectus carinatum, une hyperlaxité ligamentaire prédominante au niveau des membres supérieurs et des pieds bot varus équin. L’échographie cardiaque n'a pas objectivé d'anomalies.

**Figure 4:**
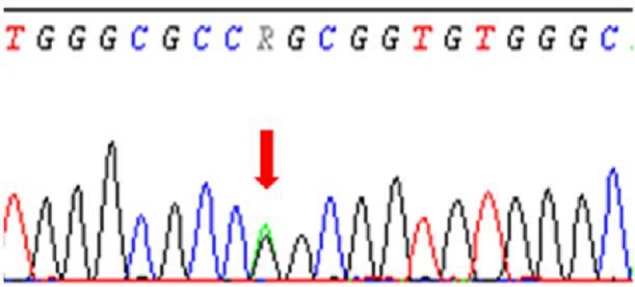
Séquence d'ADN mettant en évidence la mutation

Devant ce tableau dysmorphique, un syndrome de Costello a été évoqué.L’étude moléculaire du gène HRAS par séquençage direct de ses 5 exons, a mis en évidence une mutation faux sens c.34G>A ; p.Gly12Ser à l’état hétérozygote (Figure 4). Il s'agit d'une mutation commune du syndrome de Costello. Cette mutation n'a pas été retrouvée chez les parents, donc il s'agit vraisemblablement d'une mutation de novo.

Pour le conseil génétique, nous avons rassuré la famille de la patiente pour les prochaines grossesses. Notre patiente bénéficie d'un suivi multidisciplinaire entre pédiatres, chirurgiens orthopédiques et généticiens. Une chirurgie pour la correction de ses pieds bots varus équins a été faite et une kinésithérapie est bien suivie.

## Discussion

Le syndrome de Costello est un syndrome génétique rare, décrit pour la première fois en 1971 par Costello [3]. Son diagnostic repose en premier plan sur la clinique. Une notion d'hydramnios au cours de la grossesse, une macrosomie à la naissance et des épisodes d'hypoglycémie sont souvent retrouvées [4]. Le retard de croissance postnatal est très marqué, il est en rapport avec des difficultés alimentaires durant les premiers mois de vie [5]. Une dysmorphie faciale est spécifique à ce syndrome, elle comporte une macrocéphalie, un épicanthus, un strabisme, une myopie, une racine du nez aplatie avec un petit nez bulbeux, des oreilles bas implantées, une macroglossie avec des anomalies dentaires et un cou court. On retrouve également une atteinte cutanéo-phanérienne faite d'hyperpigmentation cutanée, d'une peau flasque, de plis profonds des mains et des pieds, d'une hyperkératose palmo-plantaire, d'une papillomatose nasale et de cheveux rares. Une hypotonie, une hyperlaxité avec des anomalies de position des pieds sont également retrouvées [6, 7]. Ces patients ont un retard du développement psychomoteur et un retard mental [8]. Une atteinte cardiaque est retrouvée dans 52% des cas, il peut s'agir d'une sténose pulmonaire, une cardiomyopathie hypertrophique ou d'une dysarythmie [1]. Ces patients ont un risque de développer des tumeurs malignes dans 15% des cas (notamment un rhabdomyosarcome et un neuroblastome chez l'enfant) [9, 10].

Le syndrome de Costello fait partie d'une classe de syndromes de développement appelée “RASopathies“. Ces derniers sont causées par des mutations germinales dans les gènes qui codent les composants des protéines de la voie de signalisation cellulaire Ras / mitogen activated protein kinase (MAPK). La Ras / MAPK est essentielle dans la régulation du cycle cellulaire, la différenciation et la croissance des cellules. Il existe de nombreux chevauchements phénotypiques entre ces syndromes (syndrome de Costello, syndrome cardio-facio-cutané et syndrome de Noonan), y compris les caractéristiques faciales, les anomalies cardiaques, les anomalies cutanées, le retard psychomoteur et la prédisposition aux cancers. Cependant chaque syndrome a des caractéristiques phénotypiques spécifiques [1, 2].

Sur le plan génétique, le syndrome de Costello est de transmission autosomique dominante. La majorité des cas sont sporadiques. Le gène responsable de ce syndrome est le gène HRAS. Il s'agit d'un proto-oncogène bien connu dont les mutations sont fréquemment retrouvées dans des tumeurs différentes. Il est localisé en 11p15.5 et comporte 5 exons [11]. La grande majorité des mutations ont été trouvées dans l′exon 1, avec une mutation rare qui a été trouvé dans l′exon 3 [1, 12].

Plusieurs séries de patients présentant un syndrome de Costello confirmé par l’étude moléculaire du gène HRAS ont été publiées [5, 7, 13, 14]. Dans ces séries, des mutations ont été trouvées dans le gène HRAS dans 85-100% des cas en fonction de la rigueur des critères du diagnostic clinique. La mutation la plus couramment retrouvée chez ces patients est la substitution p.Gly12Ser. Cette dernière a été retrouvée dans environ 80% des cas déclarés. La seconde mutation la plus fréquente (environ 9% des cas) est une substitution p.Gly12Ala. D'autres mutations ont été retrouvées chez une faible proportion de patients [9, 15]. Presque toutes ces mutations se produisent dans les résidus qui sont également connus pour être muté dans les cancers humains [7]. L'analyse moléculaire du gène HRAS chez notre patiente a trouvé la mutation commune du syndrome p.Gly12Ser à l'état hétérozygote.

Il n'existe pas de corrélations génotype-phénotype, cependant le risque de tumeurs malignes semble être plus élevé chez les individus ayant la mutation p.Gly12Ser [1, 11, 15]. Nous avons donc recommandé un suivi plus rapproché chez notre patiente.

Le conseil génétique est rassurant car la plupart des cas sont de novo. Le risque de récurrence dans la fratrie reste donc faible sauf en cas de mosaïcisme gonadique.

Il n'y a pas de traitement spécifique pour le syndrome de Costello. Pour compenser les difficultés d'alimentation, une nutrition entérale ou par sonde nasogastrique s'impose durant les premiers mois de vie. Le patient peut bénéficier d'un traitement symptomatique qui repose sur le traitement des papillomes, la correction du strabisme et de la myopie, des séances d'orthophonie, d'ergothérapie, de psychomotricité et de kinésithérapie pour les anomalies articulaires et de posture [14]. Pour dépister précocement une éventuelle tumeur (un rhabdomyosarcome ou un neuroblastome), il faut faire une surveillance médicale régulière de l'enfant ainsi que des échographies abdomino-pelvienne tous les 3 à 6 mois jusqu’à la fin de la puberté. Le pronostic dépend de la sévérité de la cardiopathie et de la survenue de tumeurs malignes.

## Conclusion

Le syndrome de Costello est un syndrome rare d'origine génétique. L'expertise clinique et l’étude moléculaire permettent de poser le diagnostic de cette maladie de la distinguer des autres diagnostics différentiels. Un diagnostic précis permet de prodiguer un conseil génétique adéquat à la famille quand à la récurrence de cette maladie chez la fratrie.

## References

[1] Gripp KW, Lin AE (2009). Costello Syndrome in: GeneReviews at GeneTests: Medical Genetics Information Resource [database online]. http://www.genetests.org.

[2] Kerr B, Allanson J, Delrue MA, Gripp KW, Lacombe D, Lin AE, Rauen KA (2008). The diagnosis of Costello syndrome: nomenclature in Ras/MAPK pathway disorders. Am J Med Genet A.

[3] Costello JM (1971). A new syndrome. NZ Med J.

[4] Lin AE, O'Brien B, Demmer LA, Almeda KK, Blanco CL, Glasow PF, Berul CI, Hamilton R, Micheil Innes A, Lauzon JL, Sol-Church K, Gripp KW (2009). Prenatal features of Costello syndrome: ultrasonographic findings and atrial tachycardia. Prenat Diagn.

[5] Digilio MA, Sarkozy A, Capolino R, Chiarini Testa MB, Esposito G, de Zorzi A, Cutrera R, Marino B, Dallapiccola B (2008). Costello syndrome: Clinical diagnosis in the first year of life. Eur J Pediatr.

[6] Quezada E, Gripp KW (2007). Costello syndrome and related disorders. Curr Opin Pediatr.

[7] Rauen KA, Hefner E, Carrillo K, Taylor J, Messier L, Aoki Y, Gripp KW, Matsubara Y, Proud VK, Hammond P (2008). Molecular aspects, clinical aspects and possible treatment modalities for Costello syndrome: Proceedings from the 1st International Costello Syndrome Research Symposium 2007. Am J Med Genet A.

[8] Axelrad ME, Nicholson L, Stabley DL, Sol-Church K, Gripp KW (2007). Longitudinal assessment of cognitive characteristics in Costello syndrome. Am J Med Genet A.

[9] Aoki Y, Niihori T, Kawame H, Kurosawa K, Ohashi H, Tanaka Y, Filocamo M, Kato K, Suzuki Y, Kure S, Matsubara Y (2005). Germline mutations in HRAS proto-oncogene cause Costello syndrome. Nat Genet.

[10] Gripp KW (2005). Tumor predisposition in Costello syndrome. Am J Med Genet C Semin Med Genet.

[11] Rauen KA (2007). HRAS and the Costello syndrome. Clin Genet.

[12] Gripp KW, Innes AM, Axelrad ME, Gillan TL, Parboosingh JS, Davies C (2008). Costello syndrome associated with novel germline HRAS mutations: An attenuated phenotype?. Am J Med Genet A.

[13] Van Steensel MA, Vreeburg M, Van Ravenswaaiji-Arts CM, Biljsma E, Schrander-Stumpel CT, van Geel M (2006). Recurring HRAS mutation G12S in Dutch patients with Costello syndrome. Exp Dermatol.

[14] Hopkins E, Lin AE, Krepkovich KE, Axelrad ME, Sol-Church K (2010). Living with Costello syndrome: Quality of life issues in older individuals. Am J Med Genet A.

[15] Gripp KW, Lin AE, Stabley DL, Nicholson L, Charles I, Scott CI, Doyle D, Aoki Y, Matsubara Y, Zackai EH, Lapunzina P, Gonzalez-Meneses A, Holbrook J, Agresta CA, Gonzalez IL, Sol-Church K (2006). HRAS mutation analysis in Costello syndrome: Genotype and phenotype correlation. Am J Med Genet A.

